# *Parisognoriste*, a new genus of Lygistorrhinidae (Diptera: Sciaroidea) from the Oise amber with redescription of *Palaeognoriste* Meunier

**DOI:** 10.3897/zookeys.50.506

**Published:** 2010-06-30

**Authors:** Vladimir Blagoderov, Heikki Hippa, André Nel

**Affiliations:** 1 Department of Entomology, The Natural History Museum, Cromwell Road, London, SW7 5BD, UK; 2 Swedish Museum of Natural History, P.O.Box 50007, S-10405 Stockholm, Sweden; 3 CNRS-UMR 7205, Entomologie, Muséum National d’Histoire Naturelle, 45 rue Buffon, F-75005 Paris, France

**Keywords:** taxonomy, new taxa, phylogeny, fossil resin, Eocene

## Abstract

A new genus and a new species of Lygistorrhinidae, Parisognoriste eocenica is described from the Eocene Oise amber of the Paris Basin. Parisognoriste sciariforme Meunier, 1904 and Parisognoriste affine Meunier, 1912 are re-described. Lectotypes are designated for both species of Palaeognoriste. The phylogenetic positions of the new genus and Palaeognoriste Meunier are discussed. The paper is an example demonstrating a new approach in cybertaxonomy including automatic generation of manuscript within Virtual Research Environment (Scratchpads), semantic enhancements, and parallel release of the publication on paper and on-line accompanied with registration of new taxa with ZooBank.

## Introduction

The Le Quesnoy locality, near Houdancourt (Oise), has yielded fossiliferous amber associated with abundant plant remains and a diverse vertebrate fauna in sediments (Nel et al. 2004). Its age is lowermost Eocene and the infrared spectra (KBr) of Le Quesnoy and Baltic ambers are very different, with that of the former more similar to the Recent Hymenaea copal. Only a small fraction of the insect genera and even fewer species of the Oise amber (also known sometimes as Paris Basin amber) are also present in Baltic amber (Nel et al. 2005). The palaeoclimate of Oise amber corresponds to the maximum global warming of the Palaeocene-Eocene boundary, which could partly explain the differences of it's fauna from the fauna of Baltic amber.

A number of specimens of Sciaroidea were discovered in the Oise amber, of which perhaps the most interesting are several specimens of a lygistorrhinid fly. The purpose of the present paper is to describe this new taxon and discuss its systematic position. Superficially, it is similar to Palaeognoriste Meunier, the two species of which were described from Baltic amber in the early 20th century. Meunier (1904) described the first, Palaeognoriste sciariforme, from the Königsberg collection of Baltic amber, based on a male and a female found in two separate pieces of amber, and erected a new genus, Palaeognoriste, to accommodate it. Later, he described another species, Palaeognoriste affine, based on two specimens found in copula in a single piece of amber (Meunier 1912). Unfortunately, both descriptions were very short and vague. To adequately describe the new genus from the Oise amber and to compare it with known fossil and recent taxa it was necessary to re-describe both species of Palaeognoriste and designate lectotypes for both species.

## Material and methods

The piece of Oise amber containing three female specimens of the new species was cut in two for better observation and polished on a slab of diatomite. Type material of both species of Palaeognoriste was also studied. Fortunately, the specimens were not lost during World War II, as many of Meunier’s other types were. The type of Palaeognoriste affine was kept in Geowissenschaftlisches Zentrum der Georg-August-Universität, Göttingen. In 2007, VB found syntypes of Palaeognoriste sciariforme in the Laboratory of Entomology, Muséum national d'Histoire naturelle, Paris, where they had been borrowed by the late Prof. L. Matile several years previously. Digital photography was undertaken using Zeiss Axioskop compound microscope and Canon EOS450D camera, the resulting images then being combined to increase depth of field using Helicon Focus v. 4.77 software. All images are available at Fungus Gnats Online website (www.sciaroidea.info).

The descriptions of new taxa and redescription of known species were prepared on the Fungus Gnats Online Scratchpad as an initial stage of testing online taxonomic workflow as described in Blagoderov et al. (2010). The paper has been semantically tagged and enhanced using the Pensoft Mark Up Tool (PMT) which is based on the US National Library of Medicine’s DTD (Document Type Definitions) TaxPub extension (http://sourceforge.net/projects/taxpub). The final XML output of the paper has been archived in PubMedCentral, a PDF uploaded in the Biodiversity Heritage Library (BHL), and all revised species registered in ZooBank (Penev et al. 2010).

## Systematics

### Parisognoriste gen. n.

urn:lsid:zoobank.org:act:D95A5DF3-9A27-474D-A504-AE90BD99F388

#### Type species.

Parisognoriste eocenica, sp. n.

#### Diagnostic description.

 Small lygistorrhinid flies. Proboscis small, about 1/3 of the height of head, palpus four-segmented, much longer than proboscis. Ocelli three, median ocellus smaller than the lateral ocelli. Scutum moderately convex, laterotergite bare. Vestiture of tibiae in rows on apical part. Outer tibial spurs 2 and 3 shorter than inner. Hind leg much longer that fore and mid leg, but its tibia and tarsus only slightly expanded. Wing membrane without macrotrichia. Sc joining C. R1 short, approximately half of wing length. Rs distinct. Crossvein m-cu present, aligned with r-m. M1 and M2 fork base and M stem weak or reduced. M3+4 and CuA without a common stem. R1 setose, Sc, R5, M, and CuA bare.

#### Etymology.

 The genus name is compound word formed from parisos (Greek πάρǐσος, almost equal, just alike) and the genus name Gnoriste. The name is feminine.

#### Discussion.

Parisognoriste resembles Archaeognoriste from the Upper Cretaceous Burmese amber, but the latter has palpi longer than head height, frons membranous, all longitudinal wing veins setose, Sc ending free, R1 longer than half wing length, M stem and base of M1 and M2 fork distinct and the tibial spurs of equal length. Parisognoriste is similar to Palaeognoriste Meunier but the latter has a long proboscis, one-segmented palpus and club-shaped hind tibia (see also under Phylogenetic analysis).

#### Parisognoriste eocenica sp. n.

urn:lsid:zoobank.org:act:FCFCCA94-9A88-4860-BF47-BBA30B83F761

##### Material examined.

###### Holotype:

complete inclusion of female in transparent fossil resin, Oise amber, deposited in the Muséum national d’Histoire naturelle, Paris: MNHN A32914 (PA876).

###### Type locality

France: Oise department: region of Creil, Chevrière, farm Le Quesnoy, 49°19.533'N, 2°40.833'E. Geological horizon: the lowermost Eocene, in amber, c. -53 My, Sparnacian, level MP7 of the mammal fauna of Dormaal (Nel et al. 2004).

###### Paratypes:

two inclusions of females in the same piece of amber (during preparation the piece was cut into two), deposited in the Muséum national d’Histoire naturelle, Paris: MNHN A32914 (PA876).

##### Morphology.

###### Female.

Measurements, mm: Length total 2.22–2.48 (holotype 2.48); wing 1.83–1.91 (holotype 1.91); antenna 0.44–0.49 (holotype 0.49); palpi 0.22.

###### Head

**Figure 1 F1:**
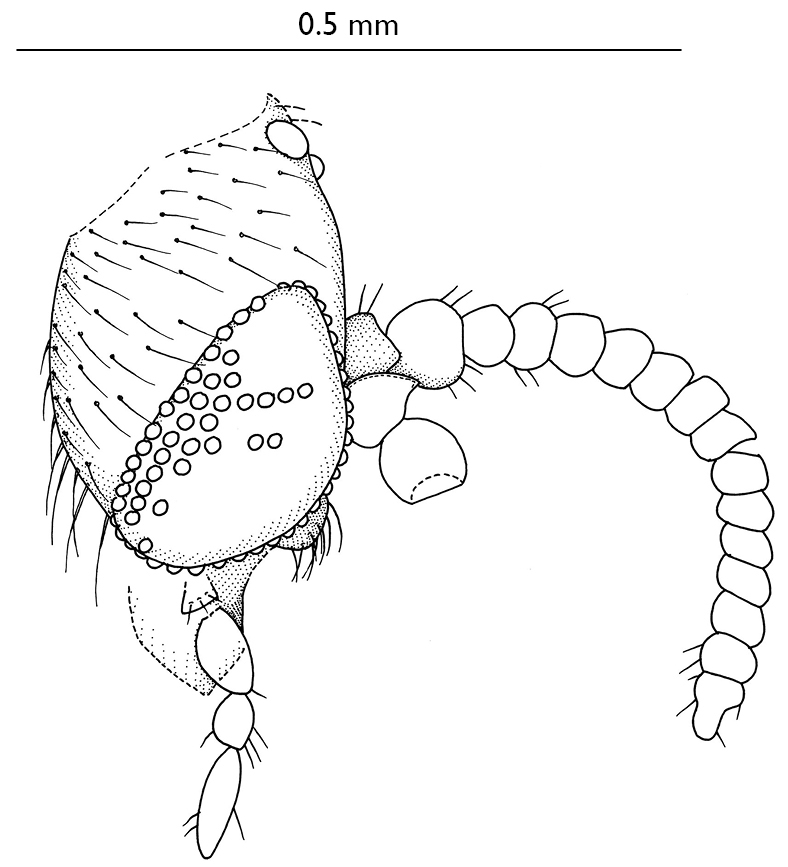
Parisognoriste eocenica, sp. n., head, lateral.

(Fig. 1) globular with short evenly distributed setae on occiput, face non-setose. Eye bare, rounded, not emarginated at antennae base, facets round and equal in size. Ocelli almost in a straight transverse line, the middle slightly smaller than the laterals, distance between lateral ocellus and middle ocellus 2.0x the distance between lateral ocellus and eye margin. Four palpomeres visible: ultimate one elongated, its length 3x the width; penultimate palpomere subglobular, slightly longer than wide; antepenultimate palpomere (the sensilliferous palpomere) about 2x longer than wide, much wider than the more apical ones; the palpomere basally from the penultimate one short, shorter than the penultimate one. Other mouthparts very short, ~ 1/3 of head height, labrum dark, triangular; labellum and hypopharynx light, narrow, sharply pointed. Antenna with 2+14 segments, apical segment secondarily divided into two. Scape and pedicel 2x wider than flagellomeres, globular. Flagellomeres subcylindrical, ca. 1.5x broader than long.

###### Thorax.

Scutum uniformly setose with lateral and posterior setae being longer. Scutellum with 10 marginal setae, which are as long and strong as the setae on the posterior part of scutum. Anteprontum with five setae, proepisternum with three setae. Pleural pit inconspicuous. Anepimerone pointed ventrally, touching katepisternum in single point. Laterotergite not produced strongly laterally, bare. Mediotergite non-setose, not convex posteriorly. Metepisternum trapezoidal, with height almost equal to width.

###### Legs.

Procoxa longer than the others. Metacoxa with a row of long posterolateral setae. Protibia about the length of profemur. Tibial organ with a small lobe, but without any distinct setation. Metatibia not conspicuously expanded apically but steadily widening from base to apex, its apical width ca. 2x the basal width. The vestiture on the apical third of tibia in rows. Spur formula 1:2:2, mesotibia with inner spur 2x the outer, metatibia with inner spur 2.5x the outer. Claws 1–3 pointed.

###### Wing

**Figure 2:**
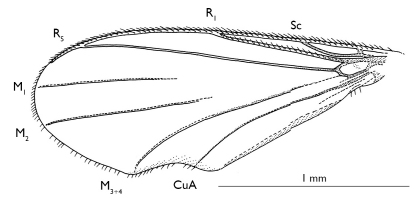
Parisognoriste eocenica, sp. n., wing

(Fig. 2). Costa extending beyond R5 tip at 3/5 distance between tips of R5 and M1. Sc joining C. Rs distinct, oblique. M stem and base of M1+2 fork inconspicuous. M3+4 reduced at base. Transverse vein connecting Rs and CuA with a distinct kink at the base of M3+4. R1 with 18–20 dorsal setae, Sc, R5, M, and CuA bare.

###### Abdomen.

**Figure 3:**
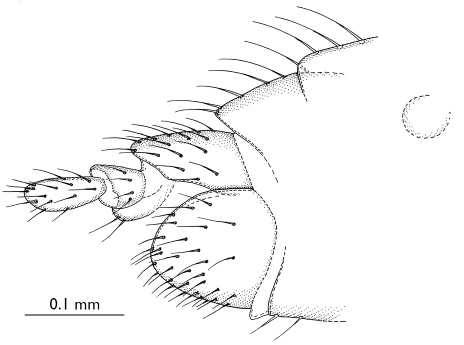
Parisognoriste eocenica, sp. n., female genitalia, lateral

Cercus (Fig. 3) two-segmented, basal segment rounded, very short, apical segment oval, its length 2x the width. Sternite 8 wide, rounded apically.

##### Etymology.

 The species name is an adjective in the nominative singular, derived from the Eocene period, referring to the temporal distribution of the species.

##### Discussion.

 See under Parisognoriste above.

### Palaeognoriste

Meunier, 1904

urn:lsid:zoobank.org:act:8E3217CC-0700-4934-8D2F-5C1F93552006

Palaeognoriste Meunier 1904: 87; Meunier 1912: 89; Matile 1990a: 360; Matile 1990b: 366, 373-376, 383, 409, 421, 554; Hoffeins and Hoffeins 1996: 311; Grimaldi and Blagoderov 2001: 55; Hippa et al. 2005: 5, 11; Blagoderov et al. 2009: 32, 33, 35, 37, 45.

#### Type species.

Palaeognoriste sciariforme Meunier 1904: 88, by monotypy.

#### Diagnostic description.

 Small lygistorrhinid flies. Proboscis medium length, about the length of fore tibia, palpus one-segmented, shorter than labellum. Three ocelli, median ocellus smaller than lateral ocelli. Scutum evenly setose with medium-length setae, laterotergite bare. Wing venation similar to Parisognoriste but Rs transverse (illustrated in Grimaldi and Blagoderov 2001: fig. 5a): Sc joining C. R1 short, approximately half of wing length. Rs distinct. R5 setose. Base of fork M1 and M2 incomplete, but traceable. M3+4 and CuA without a common stem. Crossvein m-cu well developed and aligned with crossvein r-m. R1 setose, Sc, R5, M, and CuA bare. Hind leg much longer that both fore and mid leg, hind tibia enlarged apically. Tibial vestiture in rows. Basitarsomere 3 inflated. Male tergite 9 with aggregation of thickened setae at apex.

#### Discussion.

Palaeognoriste is distinguished from all other described fossil taxa of Lygistorrhinidae by the structure of mouthparts and from all recent genera by, for example, a well-developed Rs and r-m (see below under Phylogenetic analysis).

#### Palaeognoriste sciariforme

Meunier, 1904

urn:lsid:zoobank.org:act:B8A39907-AED8-4DC1-8231-351704E7A84D

[Fig F1] Meunier 1904

Palaeognoriste sciariforme Meunier 1904: 88

##### Material examined.

 Lectotype: male, Baltic amber, Z6630, deposited in Geowissenschaftlisches Zentrum der Georg-August-Universität, Göttingen, Germany, here designated.

Notes. Meunier (1904) described the species based on two specimens, a male and a female, in two separate pieces of amber. The present study revealed that they were not conspecific. For the sake of nomenclatural stability, we designate the male specimen, Z6630, as lectotype. The female specimen, Z5125, most probably belongs to Palaeognoriste affine (see under Palaeognoriste affine). Both specimens come from the Königsberg collection of Baltic amber, which was moved after World War II to Geowissenschaftlisches Zentrum der Georg-August-Universität, Göttingen (and currently on loan in the Laboratory of Entomology, Muséum national d'Histoire naturelle, Paris).

##### Morphology.

 Male. Measurements, mm: Length total 3.8; wing 2.5; antenna 0.75; labellum 0.95; palpus 0.5.

Head rounded, dichoptic, posteriorly with a row of short (40 μm) postoccipital bristles. Ommatidia round, equal in size. Interommatidial setae shorter than ommatidial diameter. Three ocelli, laterals 2x diameter of median, touching eye margin. Clypeus triangular, pointed. Palpus one-segmented, ~ 1/2 length of labellum, with a dorsal row of setae. Antenna 2+14 segmented. Flagellomeres slightly longer than wide.

Thorax: Scutum uniformly setose, with supraalar setae longer than others. Scutellum with four longer subapical marginal setae and irregularly positioned shorter hairs. Antepronotum with three setae. Proepisternum with five setae. Suture between anepisternum and katepisternum distinct. Anepimeron separated from katepisternum with indistinct ridge. Pleural pit distinct, cut into a dorsoventral corner of katepisternum. Laterotergite produced strongly lateroventrally, with a row of seven long setae. Metepisternum trapezoidal, with stronger sclerotized anterior margin, separated from metepimeron with a strong ridge. Mediotergite evenly curved.

Legs: Metacoxa without basolateral depression. Tibial spurs 1:2:2, length 0.1: (0.25, 0.1): (0.35, 0.19), outer spur shorter. Tibial and tarsal vestiture in rows. Protibia without tibial organ. Mesotibia with a dorsal row of 7-8 dark setae on apical half, and a dense apical brush of 17-20 dark setae. Mid tarsus with a row of a few dark setae. Metatibia apically and hind basitarsomere entirely swollen. Hind tarsomeres 1–3 with strong dark ventral setae. Claws of fore and mid legs blunt, of hind leg pointed.

Wing: Costa extending 3/5 of distance between tips of R5 and M1. Sc joining C. Rs and r-m weakened, but distinct. M stem and base of M1+M2 fork inconspicuous. M3+4 base much weakened, M3+4 and CuA without common stem. Distance between apices of R5, M1, M2, M3+4 and CuA: 2.4: 1.5: 1.2: 1.8: 1.0. M1 slightly arched, M2 curved backwards apically. R1 setose, Sc, R5, M, CuA bare. Wing membrane without macrotrichia.

**Figure 4:**
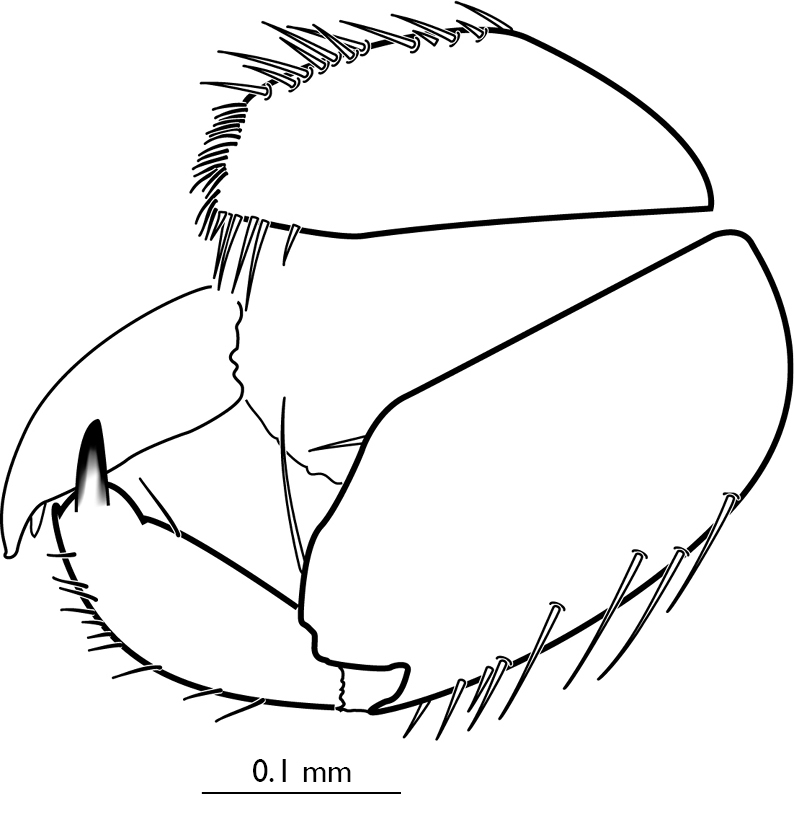
Palaeognoriste sciariforme Meunier, 1904, male genitalia, lateral

Abdomen: Tergite 9 broadly curved posteriorly, with numerous short dark spines directed posteriorly and longer light setae directed ventrally at apex. Gonostyli narrow, flattened, 4x longer than wide, each with two stout apical teeth and long dorsal mesial setae. Gonocoxite (Fig. 4) in lateral view with an obtuse angle on dorsal edge, a setae near the angle and two long setae near the base on gonostylus; covered with long setae ventrally.

#### Palaeognoriste affine

Meunier, 1912

urn:lsid:zoobank.org:act:6F42E20F-5073-473E-8463-38881FB76D10

Palaeognoriste affine Meunier 1912: 89

##### Material examined.

Lectotype: male, Baltic amber, G1848 (BST.03.382), deposited in the Geowissenschaftlisches Zentrum der Georg-August-Universität, Göttingen, Germany; here designated.

Paralectotype: female, Baltic amber, G1848 (BST.03.382), deposited in the Geowissenschaftlisches Zentrum der Georg-August-Universität, Göttingen, Germany; here designated.

Other material: female, Baltic amber, Z5125 (syntype of Palaeognoriste sciariforme), deposited in the Geowissenschaftlisches Zentrum der Georg-August-Universität, Göttingen, Germany (currently on loan in the Laboratory of Entomology, Muséum national d’Histoire naturelle, Paris).

Notes. Meunier (1912) described Palaeognoriste affine from a male and a female in copula. We here designate the male as lectotype and the female as paralectotype.

##### Morphology.

 Male. Measurements, mm: length total 3.8; wing 2.5; antenna 0.75; labellum 0.95; palpus 0.5.

Head rounded, dichoptic. Ommatidia densely set, almost hexagonal, becoming slightly smaller dorsally. Interommatidial setae inconspicuous. Three ocelli present, diameterer of medial one 0.6x of the lateral ones, lateral ocelli touching eye margin. Antennae 2+14 segmented; flagellomeres as wide as long, with one or two short dorsal setae and two short ventral spines each. Scape small, as long as wide, trapezoidal. Pedicel globular, slightly wider than flagellum. Clypeus triangular, pointed. Palpus one-segmented, 0.56x length of labellum, with a row of dorsal setae. Labellum lanceolate at apex. Hypopharynx wide and strongly sclerotized.

Thorax. Scutum uniformly setose with supraalar setae slightly longer than others. Antepronotum and proepisternum with 7–8 short setae each. Suture between anepisternum and katepisternum distinct. Pleural pit inconspicuous. Laterotergite produced strongly lateroventrally, with a row of five long setae postero-ventrally. Metepisternum trapezoidal, separated from metepimeron with a strong ridge.

Legs. Procoxa and mesocoxa with a few anteroapical setae. Metacoxa with deep lateral depression and 2 long posterolateral setae on apical 1/3. Tibial spurs 1:2:2, length 0.08: (0.17, 0.11): (0.25, 0.12), outer spur shorter. Protibia without tibial organ. Tibial and tarsal vestiture in rows. Metatibia gradually expanding apically, with a row of 10 long dorso-lateral setae and 7–8 light apical inner setae not arranged in dense brush. First tarsomere of metatarsus inflated, with nine short setae in lateral row and 14 in ventral. Claws of fore leg blunt, of mid and hind legs pointed.

Wing. Costa extends beyond R5 on 0.5x distance between R5 and M1. Sc short, joins Costa. Rs, r-m, M stem inconspicuous. Base of M1 and M2 fork traceable. M1 slightly arched, M2 almost straight apically. Ratio of distances between R1, R5, M1, M2, M3+4 and CuA 2.0:1.5:0.8:1.6:1.0.

**Figure 5:**
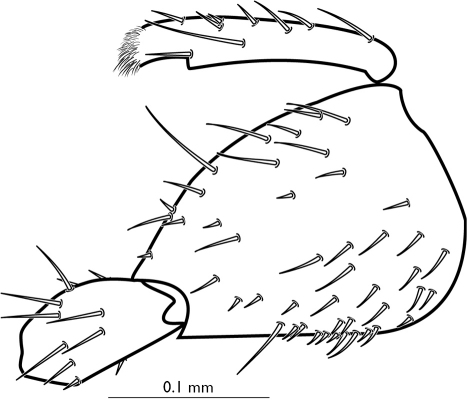
Palaeognoriste affine Meunier, 1912, male genitalia, lateral

Abdomen. Tergite 9 with a dense patch of short setae apically, without longer light setae. Dorsal margin of gonocoxites in lateral view evenly curved. Gonocoxites and gonostyli covered with long setae laterally; dorsal edge evenly curved (Fig. 5).

Female 1 (paralectotype). Measurements, mm: length total 3.8, wing 2.5, antenna 0.75, proboscis 0.95, palpus 0.5. As male, except: Compound eye smaller, not reaching lateral ocelli. All ommatidia equal in size to dorsal ommatidia of male. Interommatidia setae slightly longer than diameter of ommatidia. Ocelli in wide triangle, diameter of lateral ocellus 2x the medial ocellus, distance from lateral ocellus to eye slightly longer than to medial ocellus. Scape a little smaller than pedicel, trapezoidal. Pedicel wide, trapezoidal, 2x the width of flagellum; flagellomeres slightly shorter than wide, with numerous irregularly placed setae. Palpus 0.53x the labellum. Labellum widely rounded apically. Hypopharynx weak. Scutellum with 4 pairs of long subapical marginal setae. Antepronotum and proepisternum with 3–4 long setae each. Procoxa with long anterior setae, mesocoxa and metacoxa with long lateral setae on apical half. All claws pointed. Setae on fore basitarsomere shorter and less numerous than in male. Hind tibia with a 13–15 thin dorsal setae. Tibial spur lengths 0.11: (0.22, 0.12): (0.27, 0.13). Cercus two-segmented, basal segment globular, apical obovate, length 2x the basal one, with a few short setae laterally and ventrally.

Female 2 (syntype of Palaeognoriste sciariforme, Z5125, see under Palaeognoriste sciariforme). Measurements, mm: length total 2.9; wing 2.0; antenna 0.55; proboscis 0.75; palpi 0.4. Lateral ocelli almost touching compound eye. Flagellomeres shorter than wide. Pedicel large, round. Hind tibia with an anteriodosral row of seven thin dark setae. Hind basitarsomere with a few weak dark setae. Laterotergite with four long setae. M1 arched, M2 almost straight apically. Base of M1 and M2 fork apparent, slightly proximad of level of R1 tip. Antepronotum with 4-5 short setae, proepisternum with seven setae. All claws pointed. Cercus two-segmented, length of apical segment 2x the basal one, basal segment trapezoidal, apical elliptic with numerous long setae at apex.

##### Discussion.

Palaeognoriste affine differs from Palaeognoriste sciariforme in having the male mid tarsal claws pointed, not blunt, base of M fork weak but distinguishable, M2 straight, not curved, at apex, weaker and fewer ventral setae on hind tarsomeres 1 and 2 (11 and four pairs contra 16 and six), dorsal margin of gonocoxites evenly curved, gonocoxites setose dorsally as well as ventrally, tergite 9 without longer apical setae. It is possible that the female specimen Z5125 is not conspecific with the paralectotype of Palaeognoriste affine. However, we consider the differences rather small, and they may be a result of post-embedding distortion, and until new material become available we prefer to refer the specimen to Palaeognoriste affine.

## Phylogenetic analysis

Phylogenetic analysis was based on characters from Blagoderov et al. (2009). The data matrix was created and edited in Mesquite ver. 2.6 (Maddison and Maddison 2009) and analysed in WinNona version 2.0 (Goloboff 1999). The search parameters used were ’hold100000; hold/1000; mult*1000; mult*max’. All characters were equally weighted and multistate characters treated as non-additive. Cladograms and character distribution were analysed in WinClada verson 1.00.08 (Nixon 2002).

**Figure 6:**
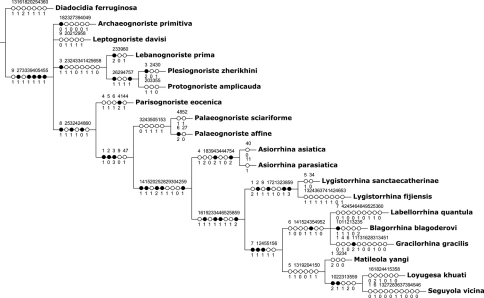
Strict consensus cladogram of four most parsimonious cladograms (176 steps, CI = 0.43, RI = 0.57). Black dots = unique character changes, open circles = homoplastic changes

Cladistic analysis yielded four equally most parsimonious cladograms of 174 steps (CI = 0.44, RI = 0.58). The strict consensus tree (Fig. 6) is congruent with previous studies (Hippa et al. 2005, Blagoderov et al. 2009). Parisognoriste forms a monophyletic group together with the clade comprising Palaeognoriste + recent Lygistorrhinidae; this group has the following synapomorphies: medial ocellus smaller than lateral (char. 8), proximal position of Rs base (char. 25), and non-setose R5 (char. 32). The taxa of the clade of Palaeognoriste + recent Lygistorrhinidae have long proboscis (char. 1), maxillary palpus shorter than labellum (char. 2), one palpomere (char. 3), ocelli almost in transverse line (char. 9), and hind tibiae club-shaped (char. 47). Although both species of Palaeognoriste form a well-defined clade, the genus has no unambiguous synapomorphies, all three supporting characters undergoing similar changes elsewhere: R5 setose (char. 35); basitarsomere 3 inflated (char. 50); tarsal claws 1 blunt (char. 51); Rs base transverse (in Parisognoriste and some Cretaceous taxa – oblique).

Parisognoriste and Palaeognoriste belong to the base of a clade of modern Lygistorrhinidae, different from the Mesozoic taxa. This replacement corresponds to significant changes of environment in the Upper Cretaceous and Palaeocene. Thus, we can hypothesise that gradual increase on length of the proboscis in Cainozoic taxa of Lygistorrhinidae reflects wider distribution of nectar-bearing angiosperms in Palaeocene-Eocene.

## Supplementary Material

XML Treatment for Parisognoriste

XML Treatment for Palaeognoriste

## Supplementary material

The Scratchpad version of this publication is available on the Fungus Gnats Online website at:

Character matrix used for the analysis is available on Fungus Gnats Online website at:

Additional images:

Parisognoriste eocenica

Parisognoriste affine

Parisognoriste sciariforme
